# 5-Aminolevulinic acid-mediated photodynamic therapy in combination with kinase inhibitor lapatinib enhances glioblastoma cell death

**DOI:** 10.1007/s10495-024-02012-w

**Published:** 2024-08-27

**Authors:** Sharayu Chandratre, Daniel Merenich, Kenneth Myers, Bin Chen

**Affiliations:** 1 Department of Pharmaceutical Sciences, Philadelphia College of Pharmacy, Saint Joseph’s University, Philadelphia, PA 19104 USA; 2 Department of Biology, College of Arts and Sciences, Saint Joseph’s University, Philadelphia, PA USA; 3 Department of Radiation Oncology, Perelman School of Medicine, University of Pennsylvania, Philadelphia, PA USA

**Keywords:** 5-Aminolevulinic acid (ALA), Protoporphyrin IX (PpIX), Photodynamic therapy (PDT), Lapatinib, Apoptosis, Necrosis, ABCG2 (ATP-binding cassette super-family G member 2), Glioblastoma (GBM)

## Abstract

5-Aminolevulinic acid (ALA) is an intraoperative imaging agent approved for protoporphyrin IX (PpIX) fluorescence-guided resection of glioblastoma (GBM). It is currently under clinical evaluation for photodynamic therapy (PDT) after the completion of GBM surgery. We previously showed that lapatinib, a clinical kinase inhibitor of epidermal growth factor receptor 1 & 2 (EGFR and HER2), enhanced PpIX fluorescence in a panel of GBM cell lines by blocking ABCG2 (ATP-binding cassette super-family G member 2)-mediated PpIX efflux, which suggests its potential for improving ALA for GBM surgery and PDT. Here we show that lapatinib enhanced PDT-induced cytotoxicity by promoting GBM cell death with the induction of apoptosis followed by necrosis. While the induction of tumor cell apoptosis was massive and rapid in the H4 cell line with no detectable Bcl-2 and a low level of Bcl-xL, it was delayed and much less in extent in A172, U-87 and U-118 cell lines with higher levels of pro-survival Bcl-2 family proteins. Lapatinib treatment alone neither reduced GBM cell viability nor had any significant effect on EGFR downstream signaling. Its enhancement of ALA–PDT was largely due to the increase of intracellular PpIX particularly in the mitochondria, resulting in the activation of mitochondria-mediated apoptosis in H4 cells. Our present study demonstrates that lapatinib inhibits ABCG2-mediated PpIX efflux and sensitizes GBM cells to ALA–PDT by inducing tumor cell death.

## Introduction

Glioblastoma (GBM) or grade IV glioma is a formidable disease with high morbidity and mortality. The current standard treatment for newly diagnosed GBM is maximum tumor resection followed by radiotherapy with concurrent temozolomide-based chemotherapy [1]. However, despite such an intensive treatment regimen, the median survival for GBM patients is only about 15 months, which has not changed over the past 20 years. There is an urgent need for improving the current standard of care and developing new therapeutic strategies to address this dismal disease outcome.

As the first choice of therapy, GBM tumor surgery aims to delay disease progression by achieving maximal removal of tumor tissues. In fact, the extent of GBM tumor resection has been shown to directly affect treatment outcomes with maximum safe tumor resection leading to better patient survival [2]. To facilitate maximum safe GBM resection, 5-aminolevulinic acid (ALA) has been approved by the FDA as an intraoperative probe for fluorescence-guided tumor resection [3]. As a small-molecule amino acid with no fluorescence on its own, ALA is orally administered to GBM patients and metabolized in the heme biosynthesis pathway inside the cell to produce protoporphyrin IX (PpIX) in the mitochondria, a porphyrin metabolite with fluorescence property. Increased ALA brain penetration due to compromised blood–brain barrier in GBM and tumor-associated metabolic changes in the heme biosynthesis pathway result in preferential PpIX accumulation in GBM tumor tissues [4]. Upon light activation, PpIX in tumor tissues emits red fluorescence, providing real-time guidance for the resection of GBM. In a landmark randomized phase III clinical trial, PpIX fluorescence-guided glioma resection after ALA administration led to significantly more total resection of contrast-enhanced tumor areas than conventional white light surgery, resulting in better progression-free survival in patients [5].

In addition to being a fluorophore, PpIX is also a photosensitizer that is able to produce cytotoxic reactive oxygen species following light activation [4]. The preferential accumulation of PpIX in GBM cells also makes it possible to target tumor cells with photodynamic therapy (PDT). ALA–PDT has been shown to eradicate GBM cells in vitro and in animal tumor models, and is showing great promise in clinical trials [6]. Particularly, ALA-induced PpIX fluorescence in GBM tissues and PpIX-mediated PDT activity have led to the use of ALA as a theranostic agent for an intraoperative procedure composed of GBM detection, fluorescence-guided tumor resection and PDT. Recent clinical studies have demonstrated the feasibility, safety and effectiveness of combining ALA–PpIX fluorescence-guided GBM resection with subsequent PDT of tumor bed for the treatment of either newly diagnosed [7] or recurrent GBM patients [8].

There have been extensive research efforts going on to enhance ALA for GBM tumor detection and PDT, which include refining the clinical protocol for better PpIX fluorescence visualization, developing novel devices and instrumentation for more sensitive and specific PpIX detection, and combining ALA with pharmacological agents for the enhancement of PpIX fluorescence and PDT response [4]. Agents that boost ALA–PpIX accumulation, inhibit PpIX outward transport, target tumor cell pro-survival mechanisms, and augment the immune response have all been explored with positive outcomes. The finding that PpIX is transported out of the cell (efflux transport) by ABCG2 (ATP-binding cassette super-family G member 2), but not other ABC transports, renders targeting ABCG2 an appealing approach for enhancing PpIX fluorescence and PDT [9]. Particularly, the identification of approved kinase inhibitors with ABCG2 inhibitory activities makes it possible to use clinically relevant drugs for the therapeutic enhancement of ALA.

Lapatinib, a dual kinase inhibitor of epidermal growth factor receptor 1 & 2 (EGFR and HER2), is among a group of FDA-approved kinase inhibitors we have screened for enhancing ALA–PpIX fluorescence in a kidney cancer cell line with strong ABCG2 activity [10]. By blocking PpIX efflux, lapatinib also enhances ALA–PpIX fluorescence in breast, lung, pancreas, skin and GBM tumor cell lines [11–13]. In our previous study, we showed that lapatinib could increase ALA–PpIX fluorescence by several folds in some GBM cell lines [12]. However, whether lapatinib was able to enhance ALA–PDT by inducing GBM cell death was not known. Given that GBM tumors often carry EGFR mutations and are refractory to cell death induced by current conventional treatments [14], it is important to determine whether and how lapatinib in combination with ALA–PDT induces GBM cell death. The present study was carried out to investigate tumor cell death after ALA–PDT alone, lapatinib alone, and combination treatments in four GBM cell lines.

## Materials and methods

### Chemicals

ALA hydrochloride from Frontier Scientific Inc. (Logan, UT) and rhodamine 123 from Life Technologies (Grand Island, New York) were dissolved in PBS. Lapatinib was obtained from LC Laboratories (Woburn, MA) and dissolved in DMSO. Chemicals were sterilized by passing through 0.22-µm pore size filters and stored in a − 20 °C freezer. The nonidet-P-40 (NP-40) cell lysis buffer (10 ×) was prepared containing 10% NP-40 (IGEPAL CA-630, MP Biomedicals), 500 mM Tris base, and 1.5 M sodium chloride. Protease inhibitor cocktail (100 ×) includes a mixture of aprotinin (200 µg/mL), leupeptin (200 µg/mL), pepstatin A (100 µg/mL), and phenylmethanesulfonyl fluoride (PMSF, 100 mM) dissolved in ethanol. Phosphatase inhibitor cocktail (100 ×) contains a mixture of sodium fluoride (1 M), sodium orthovanadate (100 mM), and sodium pyrophosphate (1 mg/mL) in water. All chemicals were obtained from Sigma unless specified otherwise. The NP-40 lysis buffer at the working concentration (1 ×) with the supplement of working concentration of protease and phosphatase inhibitor cocktails was prepared right before cell lysis.

### Cell culture

Human GBM cell lines including A172, H4, U-87 and U-118 were obtained from American Type Culture Collection (ATCC, Manassas, VA) and cultured in DMEM medium (Corning, Manassas, VA) supplemented with 9% fetal bovine serum (Atlanta Biologicals, Flowery Branch, GA) and 1% antibiotics and antimycotics (Corning). Cells were maintained at 37 °C in a humidified cell culture incubator with 5% CO_2_. Cells were routinely checked for cell morphology and growth pattern, and were used for experiments with less than 25 passages from the liquid nitrogen storage.

### PDT treatment and cell viability assay

Glioblastoma cells were seeded in 96-well plates in complete DMEM media the day before treatments. Cells were treated with ALA (1.0 mM) alone, lapatinib (0.1 µM) alone, or ALA (1.0 mM) in combination with lapatinib (0.1 µM) for 4 h. After the 4-h incubation, the plate was treated with 5 mW/cm^2^ irradiance of 633 nm light for 10 min, resulting in a light fluence of 3 J/cm^2^. Laser light was delivered through a 600-µm core diameter optical fiber connected to a diode-laser system (High Power Devices Inc., North Brunswick, NJ). A microlens was fitted at the end of the optical fiber to provide a homogenous irradiation. Light intensity was measured with an optical power meter (Thorlabs, Inc., North Newton, NJ). Immediately after the light treatment, drug-containing media were removed and cells were incubated with fresh complete DMEM medium. Cell viability was determined with alamar blue assay (resazurin from Sigma) at 48 h after treatments. The 96-well plates were read using a BioTek Synergy microplate reader (Agilent, Santa Clara, CA) with 540/35 nm excitation and 620/40 nm emission. The fluorescence intensity of treated wells was normalized to the fluorescence of control (light only) wells to indicate cell viability after different treatments.

### Detection of apoptosis and necrosis after treatment

Glioblastoma cells were seeded in 96-well black/clear bottom plates and allowed to attach overnight. Cells were incubated with ALA (1.0 mM) alone, lapatinib (0.1 µM) alone, or ALA (1.0 mM) in combination with lapatinib (0.1 µM) for 4 h, and then treated with 3 J/cm^2^ light as described above. Immediately after the light treatment, cell culture media were removed and replaced with fresh DMEM in wells treated with ALA alone or lapatinib-containing media in wells treated with lapatinib. Apoptotic and necrotic cell death were monitored in real time at various time points after treatment using RealTime-Glo Annexin V Apoptosis and Necrosis assay kits (Promega, Madison, WI). According to the manufacturer’s instruction, the luminescence and fluorescence signals of 96-well plates were read with a microplate reader (BioTek Synergy). The luminescence signal detected the externalization of phosphatidylserine (PS) on the cell membrane, a hallmark of early apoptosis; the fluorescence signal indicated the loss of cell membrane integrity, an indicator of necrosis. The combination of luminescence and fluorescence detection enables the differentiation of early apoptosis versus late apoptosis (or secondary necrosis). The signal detected from treated cells minus the signal of control (light only) cells was obtained to indicate apoptotic and necrotic cell death induced by different treatments.

### Western blotting

Glioblastoma cells at 80–90% confluency in 100-mm cell culture dishes were treated with ALA–PDT alone, lapatinib alone, or ALA–PDT in combination with lapatinib. Cells were lysed at various timepoints after treatment using the NP40 lysis buffer supplemented with protease inhibitor and phosphatase inhibitor cocktails. Cell lysates were mixed with Western blot sample buffer, heat denatured, and separated by sodium dodecyl sulphate polyacrylamide gel electrophoresis. Separated proteins were electrophoretically transferred to polyvinylidene fluoride membranes (Millipore). The membranes were first blocked with 5% bovine serum albumin in Tris buffered saline with Tween 20 (TBST) for 1 h at the room temperature, and then incubated with the primary antibody (1:1000) in TBST at 4 °C overnight. All primary antibodies were purchased from Cell Signaling Technology (Danvers, Massachusetts), which include the antibodies for poly (ADP-ribose) polymerase (PARP, catalog No. 9542), caspase 9 (catalog No. 9508), epidermal growth factor receptor (EGFR, catalog No. 4267), phospho-EGFR (pEGFR, catalog No. 3777), phospho-S6 ribosomal protein (pS6, catalog No. 4858), Bcl-2 (catalog No. 2870), Bcl-xL (catalog No. 2764) and β-actin (catalog No. 4970). After the primary antibody incubation, blots were incubated with horseradish peroxidase-conjugated secondary antibodies (1:2000) in TBST. Immunoreactive bands on the blots were visualized after the incubation with SuperSignal West Dura extended duration substrate (Thermo Scientific) and captured with a GE Amersham Imager 600 (GE Healthcare Bio-Sciences, Piscataway, New Jersey). Band intensities were quantified with the NIH ImageJ software.

### Confocal fluorescence microscopic imaging

Cells were cultured in glass bottom cell culture dishes and allowed to attach overnight. Cells were treated with either ALA (1 mM) alone or in combination with lapatinib (0.1 µM) for 4 h in complete DMEM. Rhodamine 123 (Rho123), a fluorescent mitochondria marker, was added to the medium with a final concentration of 250 ng/mL at 30 min prior to imaging to label the mitochondria. After the incubation period, cells were washed with PBS twice and incubated with serum free DMEM for imaging. Live cell confocal imaging was carried out with a Nikon TiE (Eclipse) confocal microscope using a 60 × 1.40 NA oil immersion objective as described previously [15]. PpIX fluorescence was visualized with 405 nm excitation and 705 ± 37.5 nm emission. Rhodamine 123 fluorescence was visualized with 488 nm excitation and 525 ± 18 nm emission. Differential interference contrast (DIC) images were captured to show the cell morphology. Fluorescence images from different channels were pseudo colored and merged to generate the composite images using the NIH Image J software. The colocalization between PpIX and mitochondrial marker rhodamine 123 images was analyzed with the JACoP (Just Another Co-localization Plugin) and indicated by the Pearson’s correlation coefficient between the two images.

### Statistical analysis

Depending on the number of variables in each dataset, either one-way ANOVA with multiple comparisons or two-way ANOVA with multiple group comparisons was used to determine the statistical difference between groups. Statistical significance was accepted at *P* < 0.05.

## Results

### ALA–PDT in combination with lapatinib significantly reduced cell viability in human GBM cell lines

**Fig. 1:**
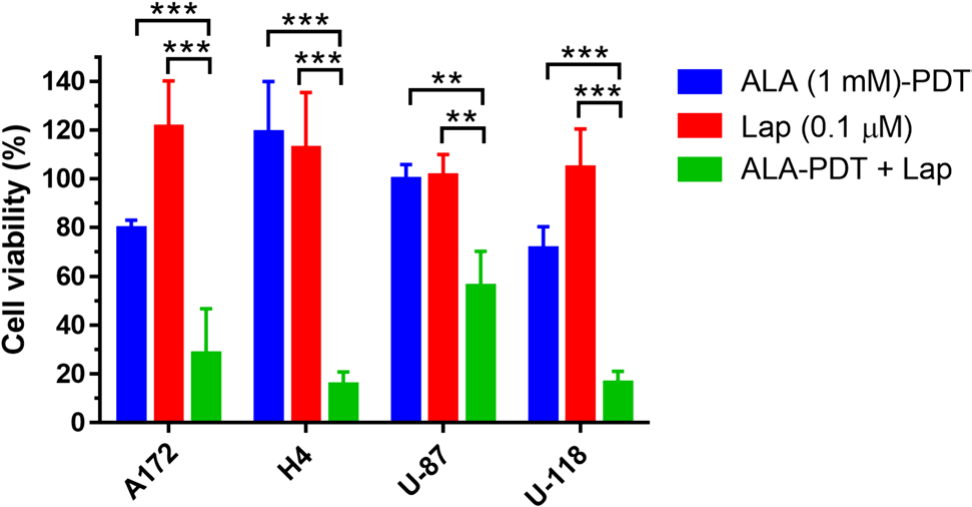
ALA–PDT in combination with lapatinib significantly reduced cell viability in GBM cell lines. A172, H4, U-87 and U-118 cells were treated with ALA (1 mM) alone, lapatinib (0.1 µM) alone, and ALA in combination with lapatinib for 4 h. Cells were then illuminated with 3 J/cm^2^ light of 633 nm and cell viability was determined with alamar blue assay at 48 h after treatment. **P < 0.01, ***P < 0.001. Experiments were repeated three times. All error bars indicate the standard deviation (SD)

Effects of ALA–PDT alone, lapatinib (4-h incubation) alone, and combination treatments on cell viability were evaluated in four human GBM cell lines including A172, H4, U-87 and U-118. ALA–PDT alone reduced cell viability to about 80% of control in the A172 and U-118 cell lines and was unable to induce any reduction in cell viability in the H4 and U-87 cell lines (Fig. 1). Incubation with lapatinib alone did not reduce cell viability in all four GBM cell lines. Compared to either ALA–PDT or lapatinib alone, ALA–PDT in combination with lapatinib significantly reduced cell viability in all four cell lines. The H4 cell line appeared to be particularly sensitive to the combination treatments, resulting in less than 20% cell viability after treatment.

### ALA–PDT in combination with lapatinib promoted GBM cell death via apoptosis followed by necrosis

**Fig. 2:**
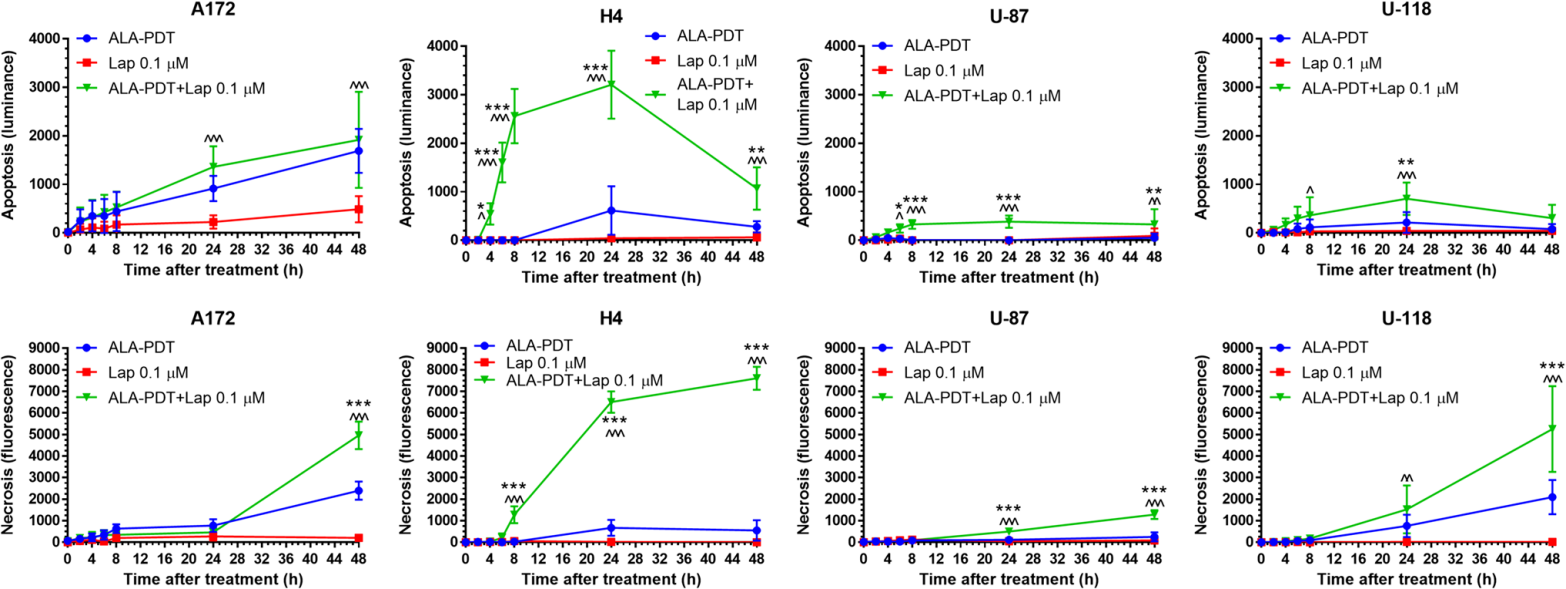
ALA–PDT in combination with lapatinib promoted GBM cell death. A172, H4, U-87 and U-118 cells were treated with ALA (1 mM)–PDT alone, lapatinib (0.1 µM) alone, and ALA–PDT in combination with lapatinib. Tumor cell apoptosis (upper panel) and necrosis (lower panel) were monitored in real time with the detection of luminescence and fluorescence, respectively, for up to 48 h after treatment. *P < 0.05, **P < 0.01, ***P < 0.001 compared with ALA–PDT alone. ^*P* < 0.05, ^^*P* < 0.01, ^^^*P* < 0.001 compared with lapatinib alone. Experiments were repeated three times. All error bars indicate the standard deviation (SD)

**Fig. 3:**
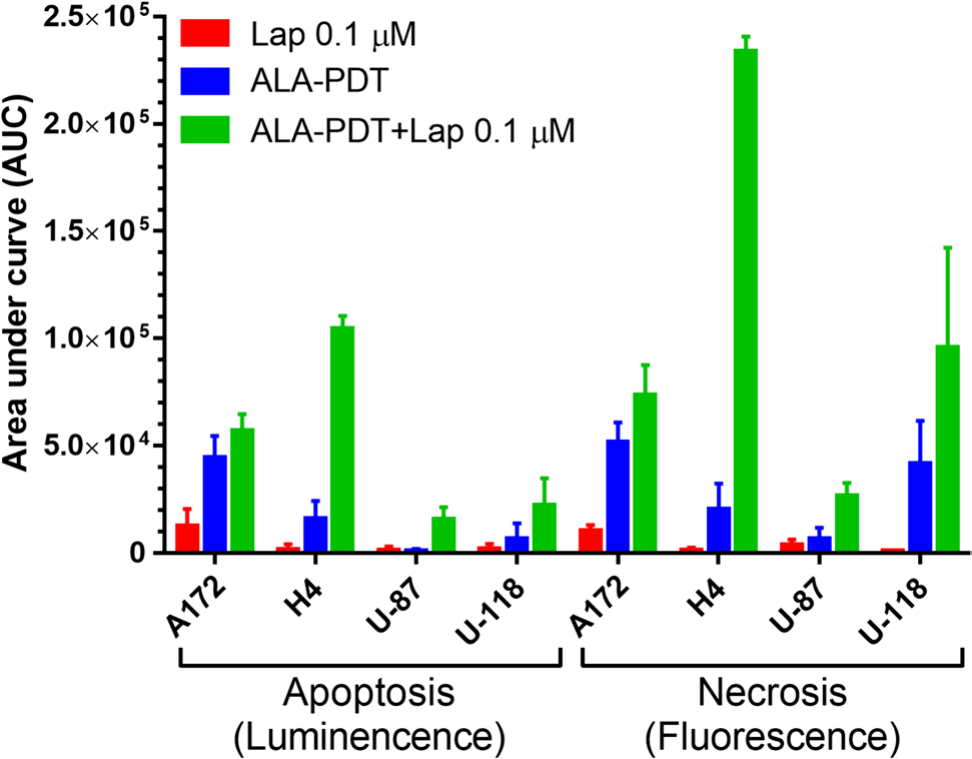
The area under curve (AUC) of Fig. 2 was calculated to indicate the mean accumulative cell death events induced by different treatments. All error bars indicate the standard deviation (SD)

To determine whether combination treatments induced GBM cell death, apoptotic and necrotic cell death was monitored in real time by luminescence and fluorescence, respectively, for up to 48 h after ALA–PDT alone, lapatinib alone, and combination treatments (Fig. 2). The area under curve (AUC) was calculated to indicate the mean accumulative cell death events after each treatment (Fig. 3). Lapatinib treatment alone did not increase apoptotic and necrotic signals in all GBM cell lines. ALA–PDT alone increased cell death events in the A172 and U-118 cell lines, but not in the U-87 and H4 cell lines. Compared to ALA–PDT or lapatinib alone, ALA–PDT in combination with lapatinib led to significant increases of cell death signals in all GBM cell lines. Particularly, the H4 cell line showed the most remarkable response to the combination treatment, which resulted in a significant increase of apoptotic signal over each individual treatment that started at the 4 h timepoint and lasted for up to 48 h after treatment. Combination treatments also significantly increased the necrotic signal in H4 cells, which was detectable at the 8 h timepoint and increased continuously to a peak level at 48 h after treatment. It was noted that the necrotic signal increase was detected at later timepoints after the increase of apoptosis signal, suggesting the occurrence of late apoptosis or secondary necrosis after treatment. Although this cell death pattern, i.e., initial apoptosis followed by necrosis, was particularly pronounced in the H4 cell line after the combination treatments, it occurred in the other three GBM cell lines as well.

**Fig. 4:**
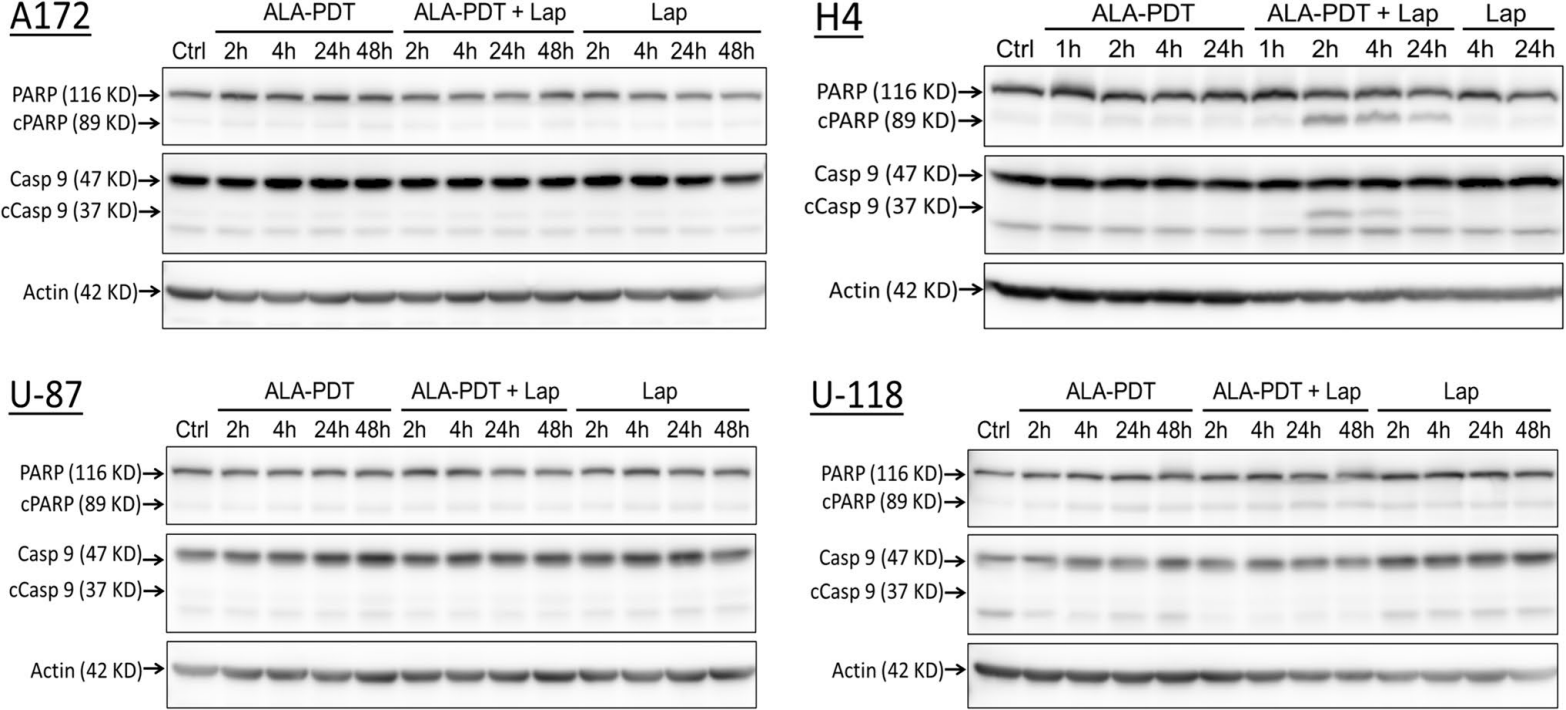
Western blot analysis of apoptotic markers in GBM cell lines after ALA–PDT alone, lapatinib alone, and combination treatments. Tumor cells were treated as indicated, lysed at various timepoints after treatment, and probed for PARP cleavage (cPARP) and caspase 9 cleavage (cCasp 9)

**Fig. 5:**
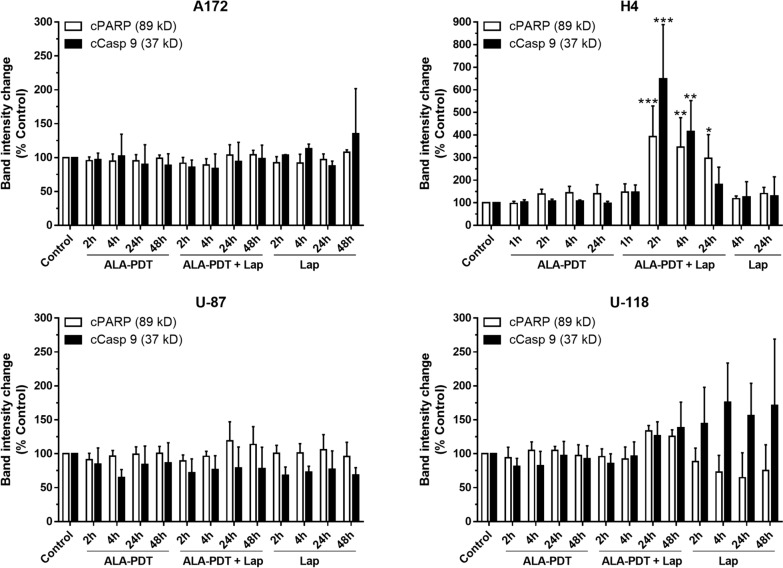
The band intensities of Western blot images in Fig. 4 were quantified and shown. *P < 0.05, **P < 0.01, ***P < 0.001 compared with the corresponding controls. Experiments were repeated three times. All error bars indicate the standard deviation (SD)

**Fig. 6:**
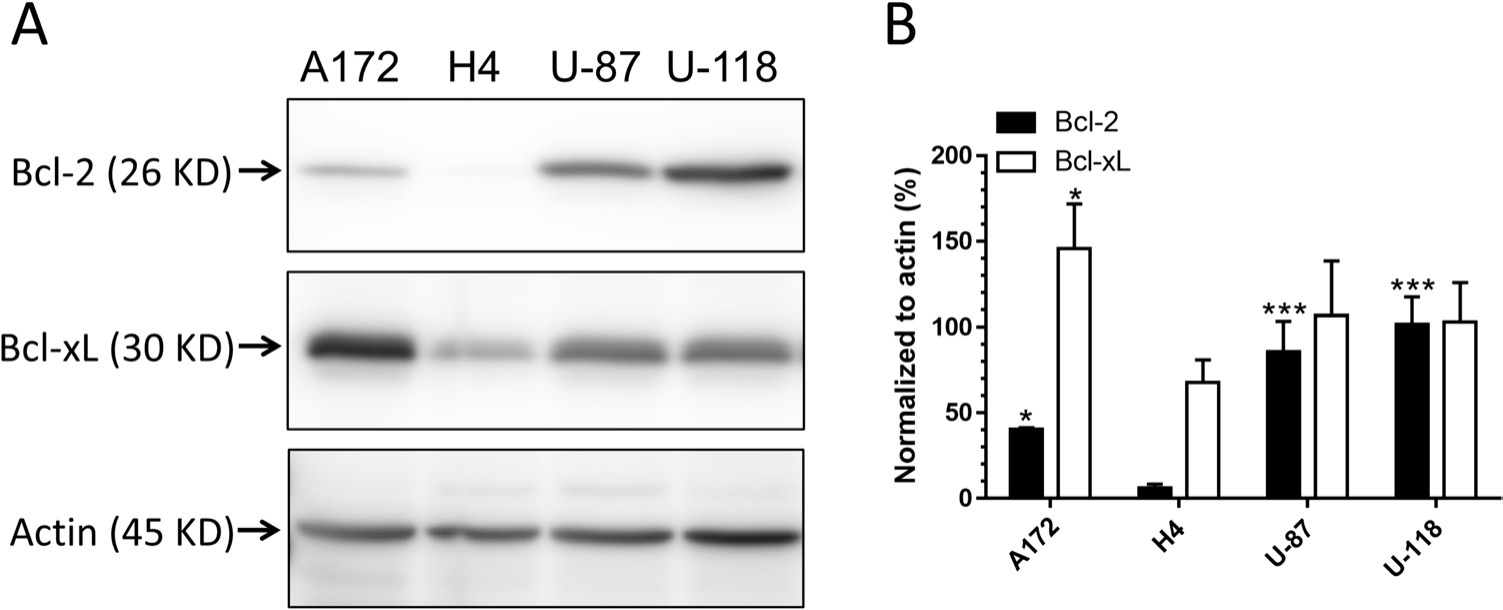
Western blot analysis of pro-survival Bcl-2 family proteins in GBM cell lines.** a** Tumor cells were lysed and probed for Bcl-2 and Bcl-xL proteins.** b** The band intensities of Bcl-2 and Bcl-xL were quantified and normalized to that of actin. *P < 0.05, ***P < 0.001 compared with the corresponding protein in the H4 cell line. Experiments were repeated three times. All error bars indicate the standard deviation (SD)

To verify the induction of apoptosis and determine the apoptotic pathway involved, tumor cell lysates were prepared at different timepoints after treatment and probed with Western blots for PARP cleavage, a hallmark of apoptosis, and caspase 9 cleavage, an indicator of mitochondria-mediated apoptotic pathway (Fig. 4). In agreement with live cell detection results (Fig. 2), ALA–PDT in combination with lapatinib induced apoptosis in H4 cells as indicated by the PARP cleavage, whereas no cleavage was detected after each individual treatment. Quantifications of band intensities showed significant increases of PARP cleavage in H4 cells after the combination treatments, but not after each individual treatment (Fig. 5). Detection of significant increases of caspase 9 cleavage in H4 cells suggests the activation of mitochondria-mediated apoptotic pathway. No significant cleavage of PARP and caspase 9 was detected after either each individual treatment or combination treatments in A172, U-87 and U-118 cell lines. To understand why less apoptosis was induced in these three GBM cell lines than in the H4 cell line, resulting in no significant cleavage of PARP and caspase 9 being detected, pro-survival Bcl-2 and Bcl-xL proteins in all four cell lines were probed (Fig. 6). Bcl-2 protein was clearly detectable in A172, U-87 and U-118 cell lines, but barely in the H4 cell line. Although all four GBM cell lines exhibited the expression of Bcl-xL, the H4 cell line showed the lowest protein level.

### Lapatinib significantly enhanced PpIX mitochondrial localization in GBM cell lines

**Fig. 7:**
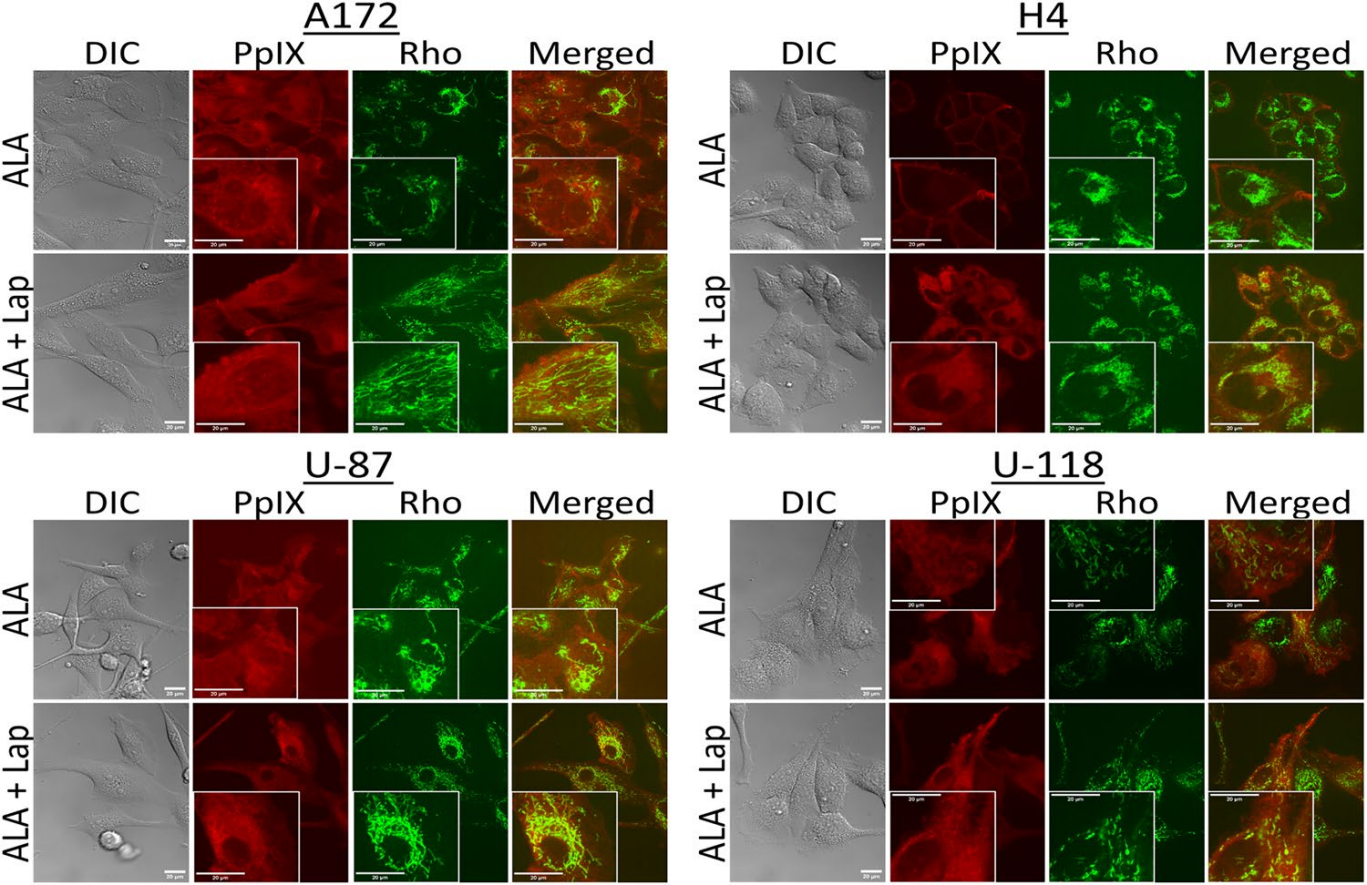
Intracellular localization of PpIX by confocal fluorescence microscopic imaging. GBM cells were treated with ALA (1 mM) alone or in combination with lapatinib (0.1 µM) for 4 h. Rhodamine 123 (Rho, 250 ng/mL) was added 30 min before imaging for labelling the mitochondria. Cells were imaged with a confocal fluorescence microscope to show the cell morphology by DIC, PpIX fluorescence in red, Rho fluorescence in green, and the merged images of PpIX and Rho fluorescence. Part of the image was magnified and shown in the insert. Bars, 20 µm

**Fig. 8:**
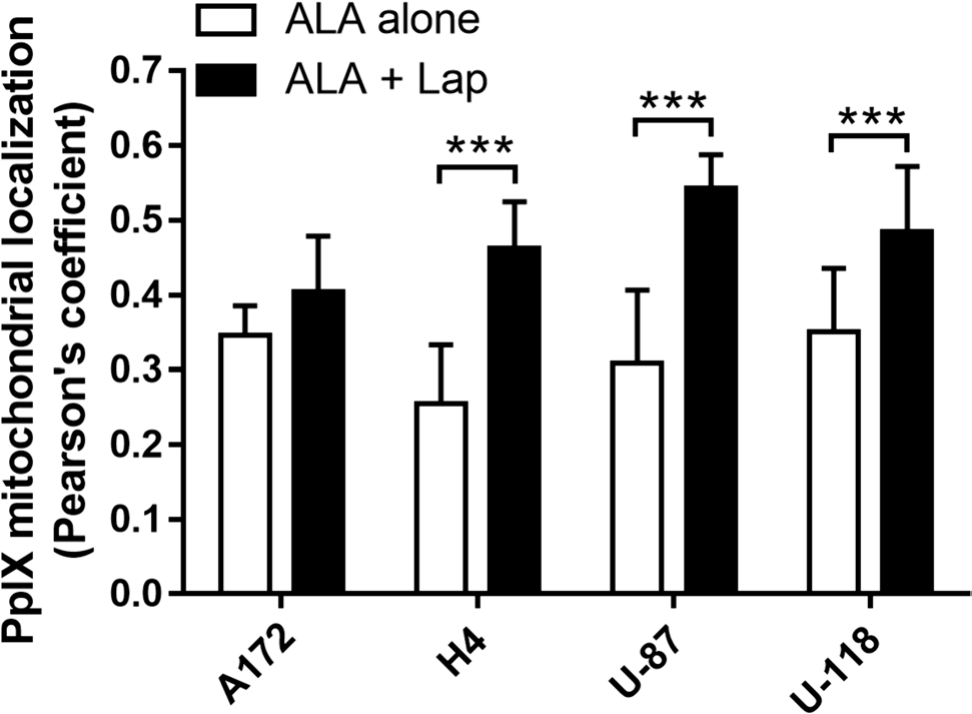
Analysis of PpIX mitochondrial localization in GBM cell lines. The colocalization between the fluorescence of PpIX and mitochondrial marker rhodamine 123 (shown in Fig. 7) was analyzed with the JACoP (Just Another Co-localization Plugin) and indicated by the Pearson’s coefficients. ***P < 0.001. Each data set contains the analysis of 10–14 images. All error bars indicate the standard deviation (SD)

The activation of mitochondria-mediated apoptosis by ALA–PDT in combination with lapatinib in H4 cells prompted the determination of PpIX intracellular localization after ALA alone or in combination with lapatinib. Confocal fluorescence imaging showed that PpIX fluorescence was very weak and predominantly at the cell membrane in H4 cells after ALA treatment alone, whereas much stronger intracellular PpIX fluorescence was detected after ALA in combination with lapatinib (Fig. 7). Colocalization analysis of PpIX and rhodamine 123 fluorescence revealed a significant increase of colocalization after combination treatments over ALA treatment alone, suggesting an enhancement of PpIX localization in mitochondria by lapatinib (Fig. 8). Significant increases of PpIX mitochondrial localization were also seen in U-87 and U-118 cell lines after combination treatments. However, no significant difference in PpIX and rhodamine 123 fluorescence colocalization was detected in the A172 cell line.

### Lapatinib inhibited EGFR phosphorylation but had no significant effect on EGFR downstream signaling

**Fig. 9:**
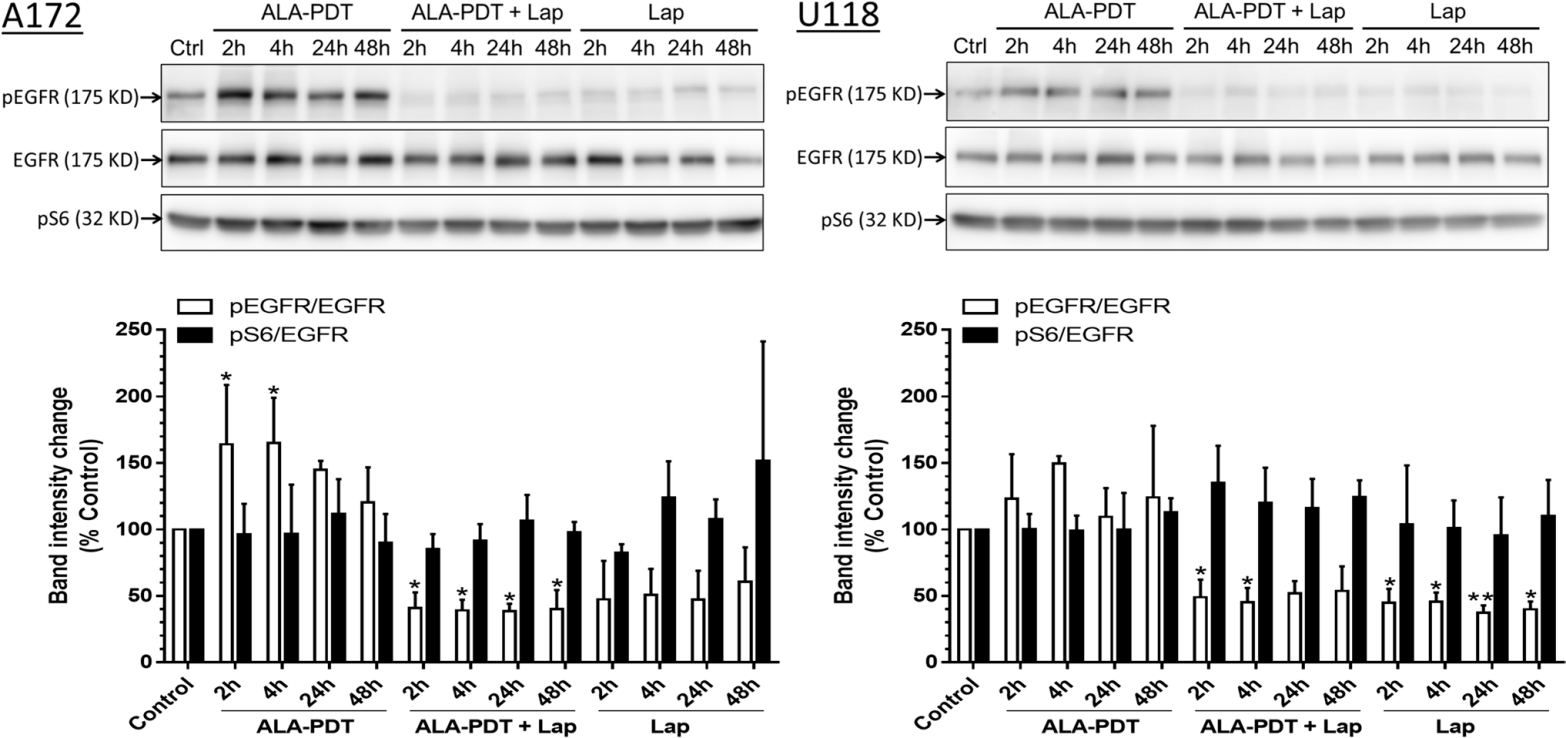
Lapatinib inhibited EGFR phosphorylation but had no significant effect on EGFR downstream signaling. Effects of ALA–PDT alone, lapatinib alone, and combination treatments on the EGFR phosphorylation and downstream signaling molecule S6 were determined by Western blots in A172 and U-118 cell lines. The band intensities of phosphor-EGFR (pEGFR) and phosphor-S6 (pS6) were quantified and normalized to the intensity of corresponding EGFR band. Experiments were repeated three times. All error bars indicate the standard deviation (SD)

Since lapatinib is an inhibitor of EGFR, which is often mutated in GBM, we examined the effects of ALA–PDT alone, lapatinib alone, and combination treatments on EGFR phosphorylation and downstream signaling molecule S6 in A172 and U-118 cell lines with detectable EGFR phosphorylation (Fig. 9). The H4 and U-87 cell lines were not included due to the negligible EGFR phosphorylation in these two cell lines. EGFR phosphorylation was upregulated after ALA–PDT alone and downregulated by lapatinib treatment alone in both A172 and U-118 cell lines. ALA–PDT in combination with lapatinib resulted in the inhibition of EGFR phosphorylation. However, neither each treatment alone nor the combination treatments had any significant effects on the phosphorylation of S6, a downstream signaling molecule of EGFR phosphorylation.

## Discussion

Following our previous study that lapatinib increased ALA–PpIX fluorescence in GBM cell lines [12], we demonstrate in the present study that lapatinib also enhanced GBM cell death after ALA–PDT. Lapatinib-induced PpIX fluorescence increase may improve ALA as an intraoperative imaging agent to achieve maximum safe GBM resection. The enhancement of tumor cell death by lapatinib would potentiate ALA as a PDT agent to eradicate tumor cells left behind after tumor surgery. This is especially important for GBM treatment because invasive GBM cells often grow diffusively at the surgical margin and even beyond, making it almost impossible to completely remove tumor cells by surgery alone [16]. Subsequent treatment of ALA–PDT in combination with lapatinib after tumor resection would help to kill the remaining tumor cells with the goal of delaying or preventing local tumor recurrence that often causes the failure of current standard treatment.

For tumor cells with innate or spontaneous resistance to ALA–PDT due to elevated ABCG2 activity, inhibition of ABCG2 activity with lapatinib was able to restore tumor cell sensitivity to PDT, making the combination treatment a necessity to obtain the therapeutic effects. This was well illustrated in the H4 and U-87, two cell lines with high ABCG2 activities as we showed previously [12]. The efflux of PpIX by ABCG2 led to low intracellular PpIX fluorescence, resulting in almost no response to ALA–PDT alone in both cell lines (Fig. 1). Suppression of ABCG2-mediated PpIX efflux by lapatinib was able to overcome this innate resistance and restore cell sensitivity to ALA–PDT. In A172 and U-118 cell lines, which exhibited limited responses to ALA–PDT alone, combination treatments led to further reduction in cell viability. These results underscore the importance and effectiveness of targeting ABCG2-mediated PpIX efflux with lapatinib for therapeutic enhancement of ALA–PDT in GBM cells.

Lapatinib-induced PDT enhancement was likely due to its effects of increasing intracellular PpIX levels and mitochondrial localization. Our previous study with flow cytometry has shown that, by blocking ABCG2-mediated PpIX efflux, lapatinib significantly increases intracellular PpIX levels in all GBM cell lines used in this study [12]. Our present work with confocal fluorescence microscopic imaging revealed that lapatinib enhanced PpIX intracellular accumulation particularly in the mitochondria (Figs. 7 and 8). PpIX produced in the mitochondria after ALA administration can be effluxed by ABCG2 localized on both mitochondria and plasma cell membrane [17]. The inhibition of ABCG2 by lapatinib resulted in more PpIX accumulation in the mitochondria, which sensitized GBM cells to PDT by activating mitochondria-mediated apoptosis. This was clearly seen in the H4 cell line in which combination treatments enhanced PpIX fluorescence in the mitochondria (Fig. 7), activated the cleavage of mitochondria-mediated apoptosis marker caspase 9 (Fig. 4), and induced more apoptosis than each individual treatment (Fig. 2). Combination treatments also increased apoptosis in A172, U-87 and U-118 GBM cell lines by live cell luminescence apoptosis measurements. However, no significant cleavage of PARP and caspase 9 was detected by Western blotting. The cause of this discrepancy was that the number of apoptotic cells induced in these three cell lines was much lower than in the H4 cell line (for the reason described below) and was likely below the detection limit of Western blotting, whereas luminescence-based apoptosis assay that captures the apoptotic signal at a single cell level has the sensitivity to detect apoptosis at such a low level.

The finding that combination treatments induced more apoptosis in the H4 than in the other three GBM cell lines raised the question of why the H4 cell line was so sensitive to the combination treatments. As we showed previously with flow cytometry measurement, U-118 actually had the highest PpIX fluorescence after ALA in combination with lapatinib, followed in order by H4, U-87 and A172 [12]. In the present study, we demonstrated that lapatinib significantly enhanced PpIX mitochondrial localization in the H4 cell line, but so did in the U-87 and U-118 cell lines. Thus, differences in the level and localization of intracellular PpIX could not explain why combination treatments induced more apoptosis in the H4 than in the other three cell lines. By probing for the pro-survival Bcl-2 family proteins, we found that the H4 cell line had no detectable Bcl-2 and a low level of Bcl-xL, whereas the other three cell lines exhibited higher levels of Bcl-xL and Bcl-2. GBM cells are known to evade apoptosis via the upregulation of pro-survival Bcl-2 family proteins [18]. The overexpression of pro-survival Bcl-2 and Bcl-xL in A172, U-87 and U-118 GBM cell lines likely protected these tumor cells from undergoing treatment-induced apoptosis as in the H4 cells despite a similar enhancement of intracellular/mitochondrial PpIX localization by lapatinib. Future efforts should focus on how to induce GBM cells with the overexpression of Bcl-2 family proteins to undergo apoptosis.

In addition to the increase of apoptosis, increased necrotic cell death was also detected after combination treatments in all GBM cell lines. Since the increase of necrotic signal lagged behind that of apoptosis, we considered this or at least some of it as the secondary necrosis (or late apoptosis) although the primary necrosis at latter times could not be excluded. ALA–PDT has been shown to induce apoptosis in some GBM cell lines [19] and it can activate tumor cell necrosis when the pro-inflammatory/survival nuclear factor-kappaB (NF-kB) pathway is inhibited [20]. It was further shown that necroptosis, a particular form of necrosis depending on the activation of receptor-interacting protein 3 (RIP3), was involved [21]. But whether and to what extent necrosis functions as a primary cell death mechanism in ALA–PDT for GBM cells remain to be determined.

Lastly, we evaluated whether the inhibition of EGFR phosphorylation by lapatinib contributed to its enhancement of ALA–PDT as lapatinib is an EGFR inhibitor and EGFR is frequently mutated in GBM tumors [14]. Effects of lapatinib in combination with ALA–PDT on EGFR signaling were evaluated in A172 and U-118, two cell lines with detectable EGFR phosphorylation that suggests the activation of EGFR signaling. Although lapatinib inhibited EGFR phosphorylation in both cell lines, it did not alter the phosphorylation of downstream signaling molecule S6, indicating that it had no significant effects on tumor cell survival driven by the EGFR phosphorylation. This finding together with the observation that lapatinib alone failed to induce cell death and inhibit cell viability in all four GBM cell lines led to the conclusion that lapatinib-induced EGFR phosphorylation had little contribution to its enhancement of ALA–PDT. These results were not surprising because current EGFR inhibitors including lapatinib, which are successful in lung cancer with activation mutations in the intracellular kinase domain, are ineffective in GBM due to the exclusive mutations in the extracellular ligand–binding domain [22, 23]. Interestingly, we noted that ALA–PDT increased EGFR phosphorylation, which was inhibited by lapatinib. Although the cause and implication of PDT-induced EGFR phosphorylation remain unclear, the finding that it did not significantly affect the downstream signaling suggests its limited effect on cell survival.

In conclusion, we found in the present study that ALA–PDT in combination with lapatinib induced significantly more cell death than each individual treatment in all GBM cell lines. Combination treatments promoted tumor cell death by inducing apoptosis followed by necrosis. Lapatinib enhanced ALA–PDT by increasing PpIX mitochondrial localization, resulting in the activation of mitochondria-mediated apoptosis. Pro-survival Bcl-2 family proteins appeared to suppress, but not completely block, the induction of tumor cell apoptosis caused by the combination treatments. Our results demonstrate that lapatinib is able to overcome GBM cell resistance to ALA–PDT alone by enhancing PpIX intracellular accumulation, mitochondria in particular, and sensitizing tumor cells to ALA–PDT by inducing cell death.

## Data Availability

Data will be available on reasonable request.
